# Whole-Body Low-Dose Multidetector-Row CT in Multiple Myeloma: Guidance in Performing, Observing, and Interpreting the Imaging Findings

**DOI:** 10.3390/life11121320

**Published:** 2021-11-30

**Authors:** Antonio Pierro, Alessandro Posa, Costanzo Astore, Mariacarmela Sciandra, Alessandro Tanzilli, Antonella Petrosino, Maria Saveria del Balso, Vincenzo Fraticelli, Savino Cilla, Roberto Iezzi

**Affiliations:** 1 Department of Radiology, “A. Cardarelli” Regional Hospital, ASReM, Contrada Tappino, 86100 Campobasso, Italy; antoniopierro.rad@gmail.com (A.P.); mariacarmela.sciandra@gmail.com (M.S.); mariasaveriadelbalso@yahoo.it (M.S.d.B.); 2 Department of Diagnostic Imaging, Oncologic Radiotherapy and Hematology, Fondazione Policlinico Universitario A. Gemelli IRCCS, L.go A. Gemelli 8, 00168 Rome, Italy; alessandrotanzilli93@gmail.com (A.T.); antonella.petrosino1990@gmail.com (A.P.); roberto.iezzi.md@gmail.com (R.I.); 3 Radiology Unit, Gemelli Molise Hospital, L.go A. Gemelli 1, 86100 Campobasso, Italy; costanzo.astore@gmail.com; 4 Hematology Unit, Gemelli Molise Hospital, L.go A. Gemelli 1, 86100 Campobasso, Italy; vincenzo.fraticelli@gemellimolise.it; 5 Medical Phisics Unit, Gemelli Molise Hospital, L.go A. Gemelli 1, 86100 Campobasso, Italy; savino.cilla@gemellimolise.it

**Keywords:** whole-body CT, low-dose CT, multiple myeloma

## Abstract

Multiple myeloma is a hematological malignancy of plasma cells usually detected due to various bone abnormalities on imaging and rare extraosseous abnormalities. The traditional approach for disease detection was based on plain radiographs, showing typical lytic lesions. Still, this technique has many limitations in terms of diagnosis and assessment of response to treatment. The new approach to assess osteolytic lesions in patients newly diagnosed with multiple myeloma is based on total-body low-dose CT. The purpose of this paper is to suggest a guide for radiologists in performing and evaluating a total-body low-dose CT in patients with multiple myeloma, both newly-diagnosed and in follow-up (pre and post treatment).

## 1. Introduction

### 1.1. Bone Marrow

Bone marrow is considered as a widespread organ (like bone, skin, and fat); it has a hematopoietic function and is therefore composed of stem cells, red blood cells, myeloid cells, and megakaryocytes: these cells perform functions of great importance, as they take part in body oxygenation, immune control, and blood clotting. These cells are surrounded by a network of cancellous trabeculae (trabecular bone or spongy bone) and lined by fibrous connective tissue. In contrast, the remaining bone marrow is occupied by fat cells. According to the percentage of its main components, the bone marrow can be distinguished in:

red (hematopoietic) bone marrow: with 60% hematopoietic cells and 40% fat cells;
yellow (fatty) bone marrow: with almost entirely (95%) fat cells.

Bone marrow composition undergoes various changes during the years, with multiple areas of hematopoietic bone marrow that begin to transform into the fatty bone marrow. This process starts at birth, going from the bone border to the central bone, in a symmetrical and centripetal fashion, following a predictable sequence during two decades. In the first ten years, bone marrow conversion begins in the long bones, starting in the diaphysis and progressing toward the metaphysis (particularly the distal metaphysis). In the second decade, the long bones’ marrow becomes mainly yellow, with the exception of residual regions of red marrow in the proximal metaphysis. In the late third decade, the bone marrow distribution becomes mature, with red marrow persisting in the axial skeleton (skull, spine, sternum, clavicles, scapulae, pelvis) and in the proximal metaphysis of the long bones [1,2,3].

### 1.2. Multiple Myeloma

Multiple myeloma (MM) is a clonal plasma cell proliferative disease characterized by primary bone marrow infiltration, which causes increased osteoclasts activity and decreased osteogenic activity, determining osteolytic lesions which eventually lead to hypercalcemia, renal failure, anemia, and/or osteolytic lesions; these manifestations are also known as the acronym CRAB (from Calcium elevation, Renal dysfunction, Anemia, Bone disease) [4,5].

MM is part of a group of pathologies defined as monoclonal gammopathies (whose classification is based both on the amount of M protein in the urine and on the plasma cell percentage), which also include the monoclonal gammopathy of uncertain significance (MGUS), smoldering MM and overt MM [4,6,7,8,9,10]. MGUS is present in about 3–4% of the global population older than 50 years, and around 20% of these patients will develop MM or related conditions (e.g., lymphoproliferative diseases, amyloidosis, etc.) [11]. Up to 80–90% of MM patients during the course of the disease will present with purely osteolytic bone lesions, usually accompanied with tough bone pain, pathological fractures, hypercalcemia, spinal cord compression, and increased mortality [12,13]. Whole-body imaging represents the standard in diagnosing MM, as recommended by the International Myeloma Working Group (IMWG): in particular, low-dose whole-body CT (WBLD-CT) examination should be performed first due to its diffuse territorial availability, fast acquisition times and low costs; whole-body or spine magnetic resonance imaging (MRI) examination should be performed in case of suspicious lesions seen at WBLD-CT that do not fulfill the diagnostic CT requirements; whole-body PET-CT examination can be helpful in previously treated patients. CT detection of one or more osteolytic bone destruction sites (size ≥ 5 mm) fulfills the requirements for multiple myeloma diagnosis [4]. Radiographic skeletal examination has been the preferred imaging technique, but it should not be used unless it is the only option, as its accuracy is very low, with a wide range (30–70%) of examinations resulting in false negatives. WBLD-CT represents a better solution for diagnosing osteolytic disease in MM patients, with greater cost-effectiveness [11,14].

## 2. WBLD-CT Protocol and Dose

**Figure 1:**
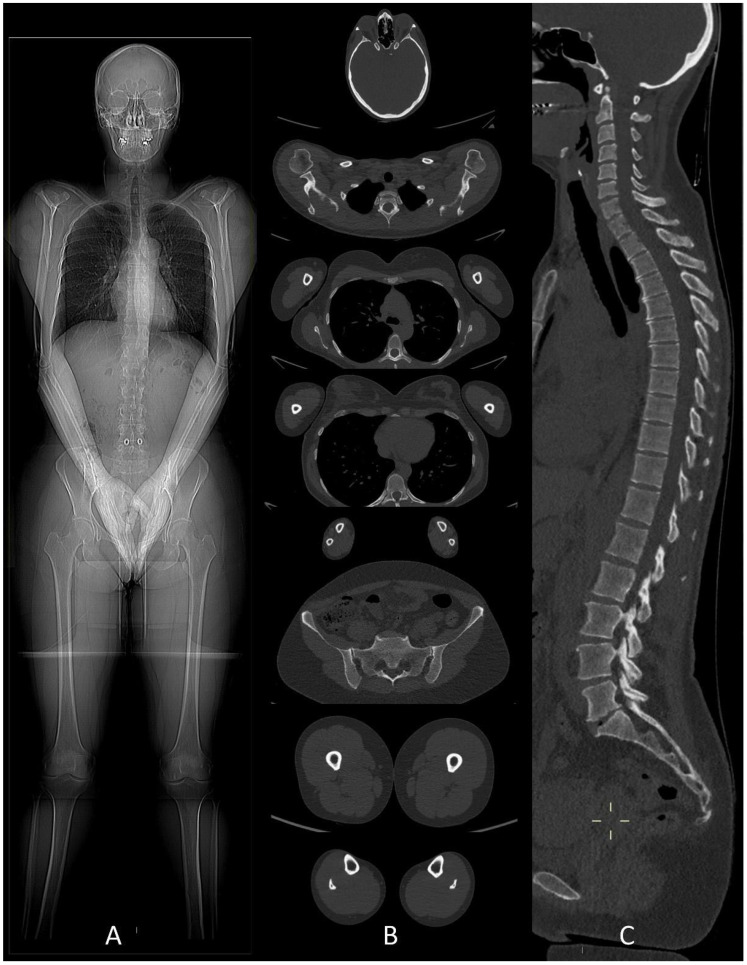
(**A**) CT scout-view showing the correct patient position in the CT scanner to avoid beam-hardening artifacts on thoracic and lumbar spine. (**B**) Whole-body low-dose CT examination performed from the skull down to the lower limbs. (**C**) Sagittal CT image showing cervical, dorsal and lumbar spine with “bone” algorithm is the best plane to identify vertebral compression fractures.

Whole-body low-dose CT examinations are performed without any preparation nor contrast agent. The patient stays in the supine position assuring that limbs are in the field of view. The upper limbs must be in front position, above the abdomen with the hands joined (Figure 1), to avoid the image having poor quality due to streak artifacts generated by beam hardening on the spine [15].

**Figure 2:**
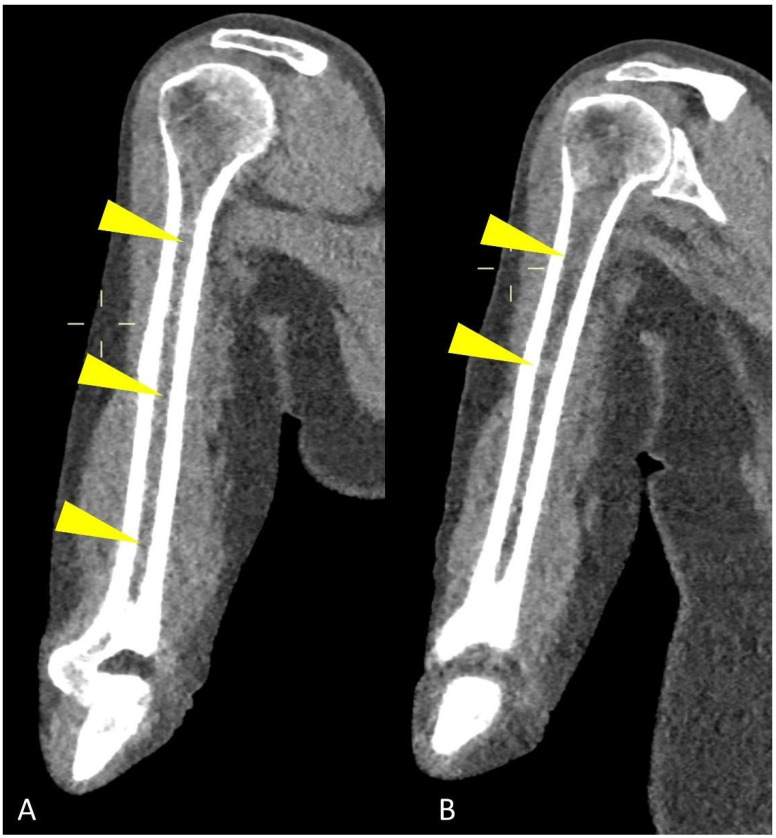
Coronal CT image showing (yellow arrowheads) humeral abnormal medullary lesions with high CT density in a patient with MM with (**A**) diffuse and (**B**) focal pattern.

Study protocol usually requires at least a 64-slices scanner CT with the following acquisition parameters: 120 kV tube voltage and 30 mAs tube current, even though protocol variations using 140 kV tube voltage with 14 to 25 mAs current showed to be effective as well [16,17]. Increased radiation intensity up to 40 or 50 mAs is suggested for overweight patients and with known or expected reduced bone density [14]. Dual-Energy CT (DECT) protocols, exploiting the different attenuation of pathological and normal bone findings at different kV tube voltages, could be helpful in differential diagnosis [18]. Rebuilding algorithms help assess the quality of bone structure and para-medullary and extra-medullary soft tissues, as well as being the most powerful solution for evaluating focal and diffuse high-density myeloma deposits in the long bone marrow cavity (Figure 2).

CT scan should start from the top of the skull down to the proximal tibial metaphysis (depending on patient’s height) [19,20]. As multiple myeloma’s osteolytic lesions can be as small as 5 mm to be considered for diagnosis, slice thickness when interpreting the axial images is advisable to be of at least 3 mm, if not 1.25 mm; 1.25 mm slice thickness is also important to correctly evaluate multiplanar reconstructions (MPR), as it grants an isotropic voxel and therefore a great spatial resolution.

## 3. WBLD-CT Imaging Findings and Evaluation

**Figure 3:**
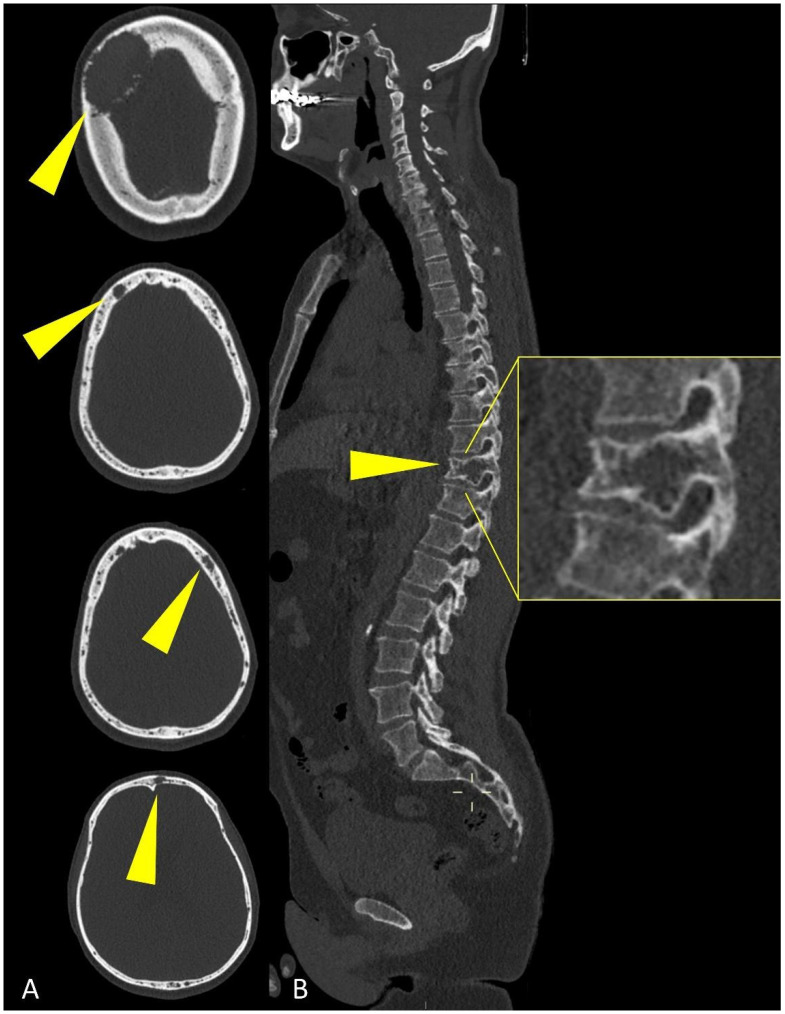
Multiplanar CT images showing (yellow arrowheads) osteolytic lesions of (**A**) the skull and of (**B**) a dorsal vertebra (detail in (**B**)).

WBLD-CT can assess MM extension better than traditional plain radiograph, because it can show both osteolytic and extra-medullary lesions (Figure 3).

Studies on total-body MRI demonstrated that imaging evaluation of the spine alone in MM patients can miss up to 50% of myeloma lesions, therefore, a WBLD-CT study must include all the parts out of the spine to obtain a correct staging and risk stratification of MM patients [21].

**Figure 4:**
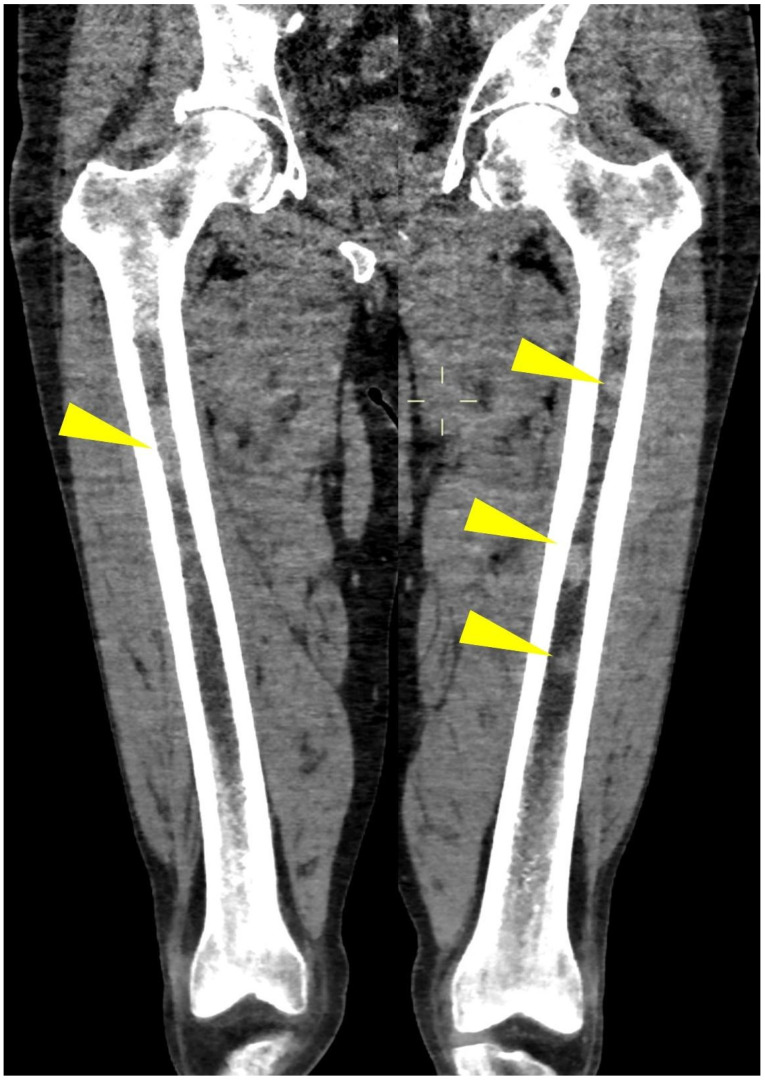
Coronal CT image of (yellow arrowheads) bilateral femoral focal intra-medullary high CT density lesions in a patient with MM.

Axial images using a “bone” reconstruction algorithm must be systematically performed, usually in a cranio-caudal fashion, starting from the skull to the cervical, thoracic, and lumbar spine, pelvic bones, and lower limbs, whereas ribs, sternum, scapulae, clavicles, and upper limbs are evaluated afterward (Figure 1B). Once completed, it is advisable to analyze the sagittal multiplanar reconstruction (MPR) images of the spine to identify vertebral fractures and to assess the risk of neural compression (Figure 1C). MPR images are also useful to differentiate benign osteoporotic vertebral fractures from malignant ones (although MRI remains the gold standard for differential diagnosis of vertebral collapse): predictive CT findings of benign vertebral fractures are the presence of fracture lines within vertebral body, retropulsion of a bone fragment from posterior wall into the spine canal, evidence of paraspinal soft tissue widening, and intravertebral “vacuum sign” defined as the presence of an air cleft in the vertebral body. Features associated with malignant vertebral fracture are the presence of extended destruction of the vertebral body (cortex and cancellous bone) and pedicles, as well as a focal soft tissue mass in the paravertebral or epidural space [15]. Focal and/or diffuse intra-medullary infiltration of femur and humerus can be found by analyzing axial and MPR images (Figure 2 and Figure 4) [22].

After bone evaluation, soft-tissue image analysis is necessary to find extramedullary disease or non-osseous incidental findings [15,23].

**Figure 5:**
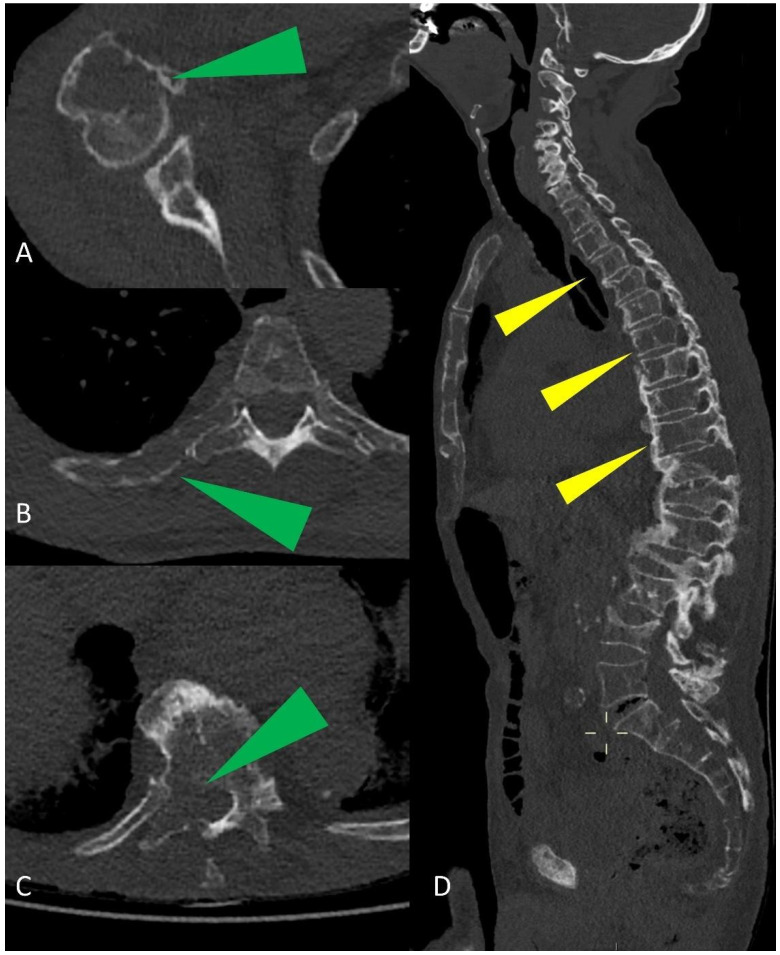
Multiplanar CT images showing humeral, ribs, and vertebral osteolytic lesions (green arrowheads in (**A**–**C**)); multiple osteolytic lesions of the spine (yellow arrowheads in (**D**)).

WBLD-CT imaging performs best in cases of diffuse osteopenia, lytic lesions, endosteal scalloping, cortical disruption and extraosseous involvement: up to 10–15% of MM patients present with diffuse osteopenia or osteoporosis at diagnosis [4,11]. Lytic bone disease is the most common feature of MM, with up to 70–80% of patients having osteolytic lesions at diagnosis, and up to 90% developing lytic lesions during the disease evolution (Figure 5), leading to pathological fractures, brutal bone pain, and hypercalcemia [24,25].

**Figure 6:**
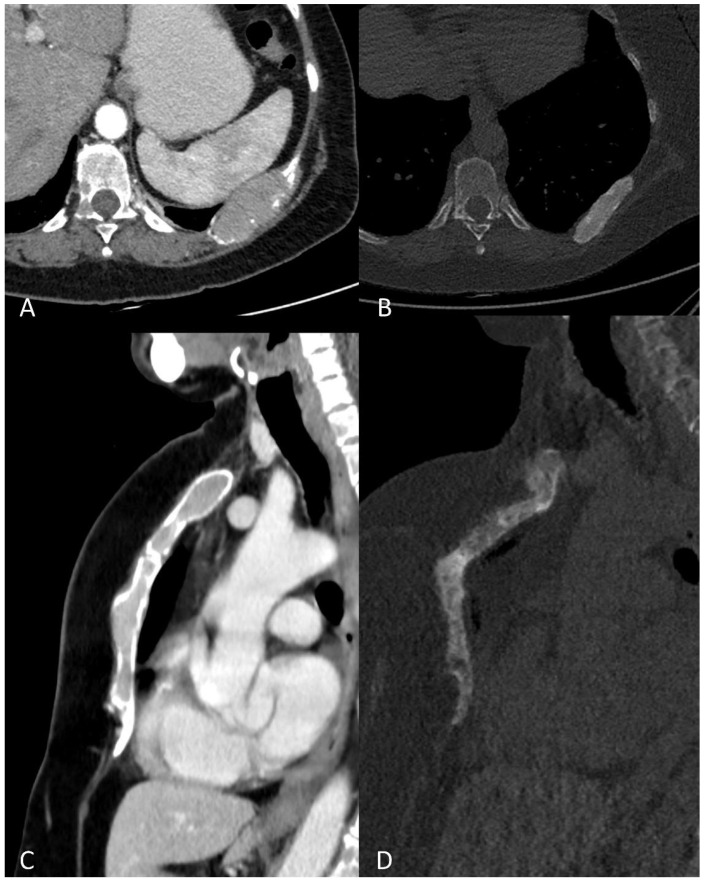
Multiplanar CT images showing (**A**) an osteolytic lesion of a rib, with (**B**) development of sclerosis and size-reduction after treatment. (**C**) Diffuse hyperdense myeloma deposits in the medullary cavities of the sternum with (**D**) development of sclerosis and size-reduction after treatment.

MM-related osteolysis is represented by small, focal, low-density lesions of trabecular bone without sclerotic boundaries (unless prior treatment) (Figure 6); these lesions are morphologically defined in literature by IMWG criteria as typical punched-out osteolytic areas with a diameter greater than or equal to 5 mm without reactive sclerosis of the surrounding bone [26].

CT is more sensitive compared to MRI in the detection of osteolytic bone lesions and better evaluates spinal stability in vertebral fractures; on the other hand, MRI is the reference standard method to detect bone marrow infiltration prior to bone fracture, as well as the various features of medullary involvement, ranging from focal and well circumscribed lesions to diffuse infiltration pattern; therefore, radiologists should always recommend MRI evaluation for those suspected small lesions that do not meet the strict abovementioned size criteria [27].

**Figure 7:**
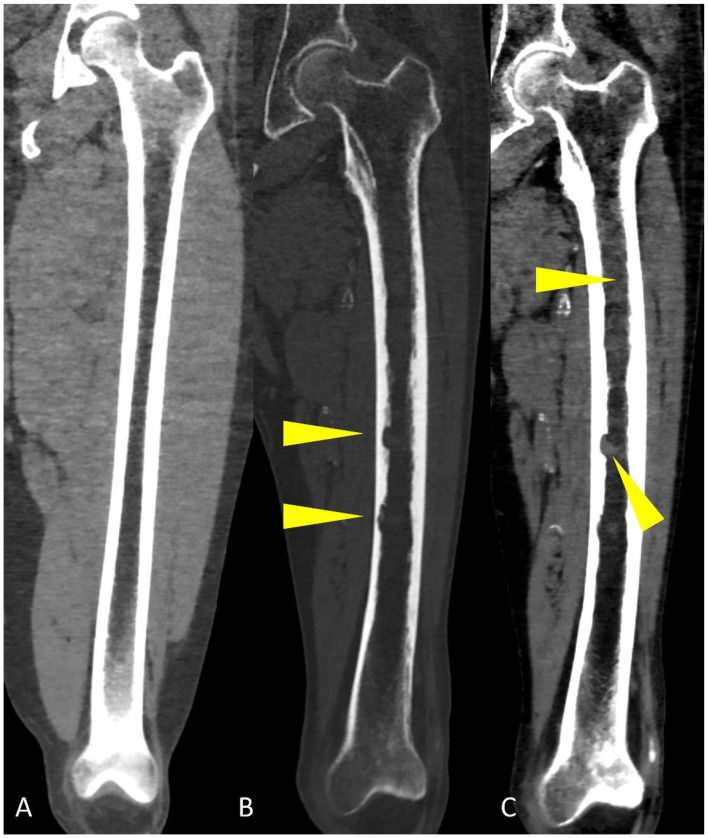
Coronal CT reconstruction images of the femoral bone showing: (**A**) regular aspect of bone marrow without osteolytic lesions, endosteal scalloping and pathological deposits; (**B**) “bone” algorithm reconstruction, used to evaluate osteolytic lesions and endosteal scalloping (yellow arrowheads); (**C**) “soft tissue” algorithm reconstruction used to assess focal and diffuse hyperdense deposits in the medullary cavities of long bones (yellow arrowheads).

WBLD-CT protocols allow identifying the lesions against well mineralized trabecular bone easily, even though, in patients with significant osteoporosis, their detection and differentiation from areas with rarefied trabeculae can be more challenging [15]. Endosteal scalloping is linked to focal resorption of the endosteum (the inner layer of the bone cortex), due to slow-growing medullary lesions (Figure 7), generating cortical thinning, more evident in the long bones.

**Figure 8:**
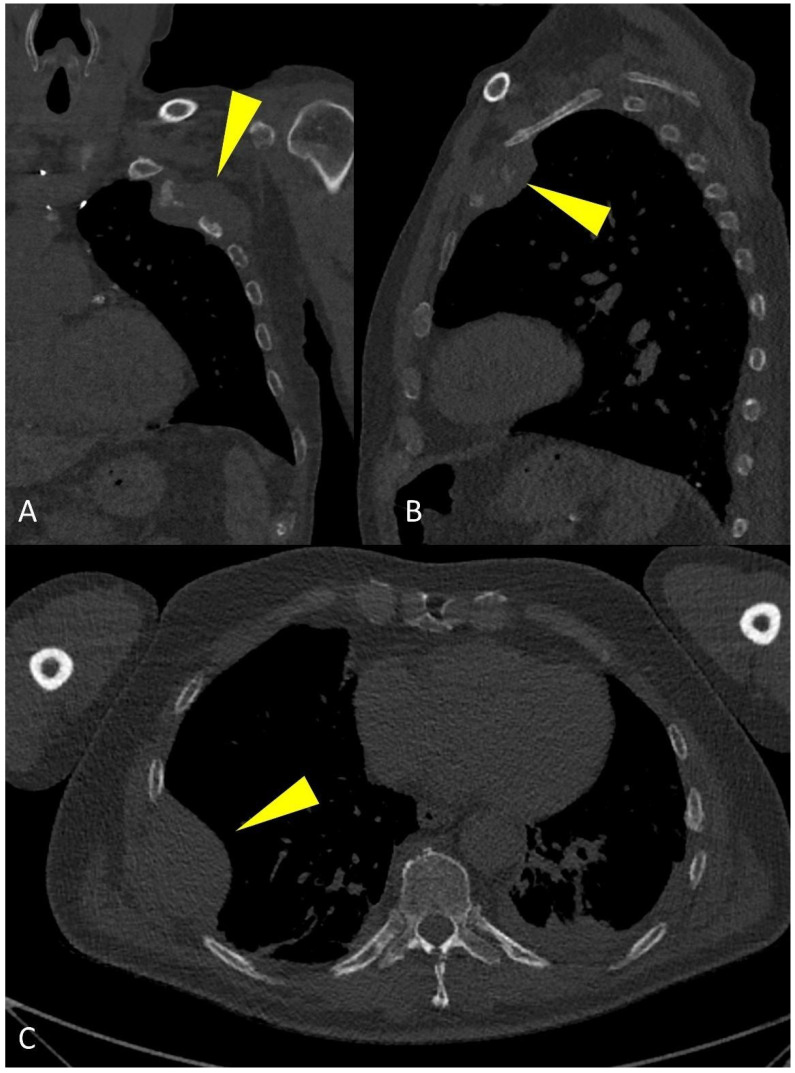
Multiplanar CT images showing a large para-medullary lesion (yellow arrowheads in (**A**–**C**)): extra-osseous pathologic tissue in the chest wall soft tissues originating linked to skeletal involvement of the rib.

In the case of para-medullary disease, soft tissue infiltration originates from a bone lesion, with the presence of extra-osseous lesions linked to skeletal involvement (Figure 8); PET-CT is the study of choice when extramedullary disease is suspected, and is also frequently used in patients with non-secretory MM [28].

Not all low-density trabecular bone lesions can be considered osteolysis: according to current literature, myelomatous lesions are those osteolytic lesions with plasma cell infiltration and a positive, tissue-like density, expressed as Hounsfield Units (HU): quantitative density measurements on WBLD-CT are necessary to distinguish between fatty hypodense bone marrow (ranging from −30 to −100 HU) and plasma cell infiltrates, which have a higher CT density (average 55 HU); moreover, the role of Dual-Energy CT (DECT) is nowadays growing in identifying bone marrow infiltration in non-osteolytic lesions, using the HU measurement references, as plasma cell infiltrates and fatty bone marrow have different attenuation values at CT acquisitions with different kVs [18,27,29].

**Figure 9:**
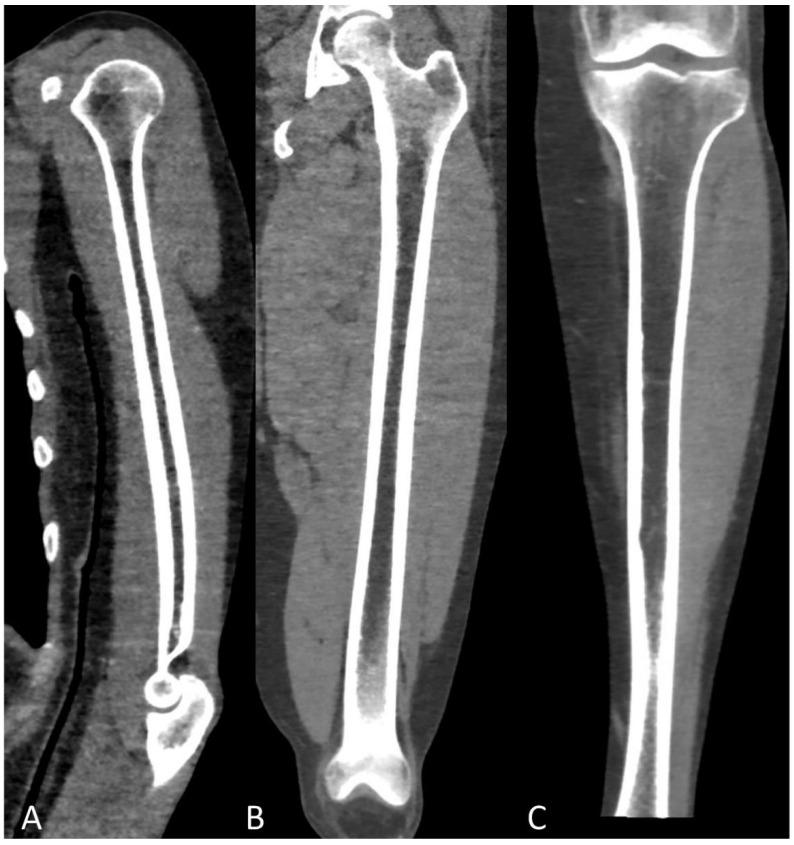
Multiplanar CT images with “soft tissue” window showing the distinctive aspect of normal (**A**) humeral, (**B**) femoral, and (**C**) tibial fatty bone marrow on low-dose CT in healthy adults.

In the long bones of adults, the physiological conversion of hematopoietic bone marrow to fatty bone marrow allows the easy detection of abnormal cell infiltration (diffuse or nodular) with WBLD-CT: most of the intramedullary space of appendicular and axial bones in healthy adults is usually replaced by fatty bone marrow (Figure 9), which in CT imaging evaluation has lower CT attenuation values than the density of water [30,31,32].

**Figure 10:**
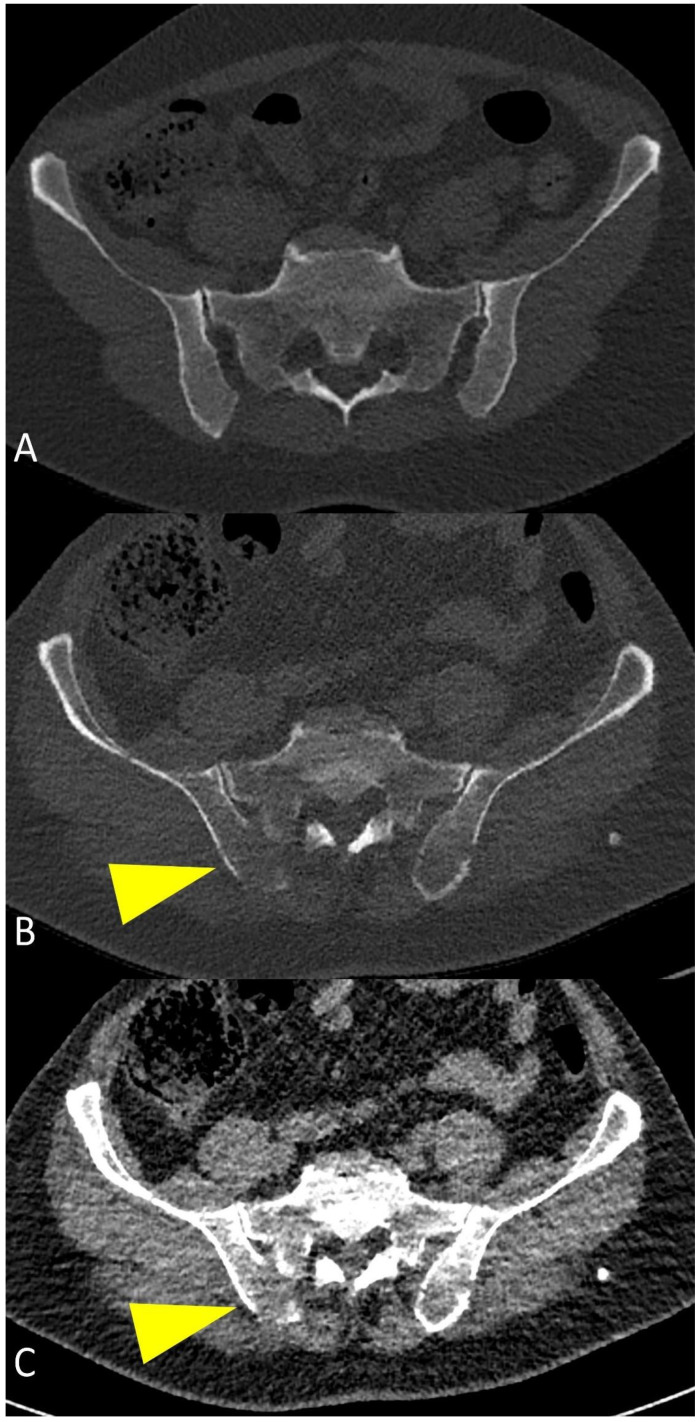
(**A**) Axial CT images showing normal appearance of bone and bone marrow in an axial skeleton with dense trabeculae. (**B**,**C**) myeloma-related osteolysis appearing as focal destructive lesions of the trabecular bone with cortical interruption and without a sclerotic border (yellow arrowheads).

When neoplastic cells, as in MM, occupy this space, due to the destruction of mineralized bone, the marrow lesion densities are characterized by solid (myelomatous) tissue that shows positive HU values (Figure 2, Figure 4 and Figure 10) [20].

It is important to emphasize that WBLD-CT has a good positive predictive value (94.1%) when osteolytic bone lesions are detected, confirming the diagnosis of MM [4]. A comparison between the WBLD-CT at baseline and after therapy is important to detect both signs of bone healing and bone destruction that are clinically relevant. Even when disease recurrence is suspected, based on serological tests, a WBLD-CT is recommended to assess the extent of bone lesions [4]. A well known disadvantage of CT examinations is represented by the use of ionizing radiation, even though low-dose protocols technical improvements, such as iterative reconstruction algorithms and dual-source CT scanners, considerably reduce the effective radiation dose [27].

On the other side, WBLD-CT imaging is not so efficient in detecting early bone marrow infiltration, when the bone destruction is not such as to cause frank osteolysis, particularly in the dense trabecular bone of spine and pelvis, as it is not easy to determine whether myelomatous cellular infiltration replaces the fatty bone marrow component, due to the CT density of these sites being a function of bone marrow composition and trabecular bone mineralization (Figure 10); in this setting, in case of negative WBLD-CT, a whole-body or spine and pelvic MRI should always be suggested in the radiological report as it is the most suitable imaging technique for patients with smoldering or asymptomatic MM, to exclude myelomatous lesions, to confirm the diagnosis of smoldering MM, or to find undetectable-CT lesions [27].

The IMWG introduced in 2014 different myeloma-defining events in order to treat patients earlier (SLiM-CRAB criteria): in particular, patients with more than one focal lesion at MRI examinations should be considered as “high-risk” patients and have indication for systemic therapy; therefore, according to this criterion, in the differential diagnosis between smoldering MM and MM a whole-body low-dose CT should be performed to rule out any osteolytic lesion and, if negative, a whole-body MRI or MRI of spine and pelvis should be performed to exclude the presence of focal myelomatous lesions [4,32].

Non-pathological foci of fat can be seen under normal conditions in various bones and could be misinterpreted as osteolytic myelomatous lesions during WBLD-CT, even though patient anamnesis can help differentiate the two entities: if a hypodense fat-containing lesion is seen in a WBLD-CT of a patient with untreated or undiagnosed MM, the lesion should not be considered as a certain pathological myelomatous lytic lesion; conversely, in a patient with treated MM, the osteolytic lesions may show partial or total fat replacement, as well as a size reduction and central or peripheral sclerosis (Figure 6), therefore, in MM patients undergoing treatment, 18F-FDG PET-CT should be used for baseline and post-therapy assessment, to evaluate treatment response [15].

Infiltration of the humeral and femoral bony canals can be classified as focal or diffuse according to the pattern of the dense area (Figure 2): the diffuse pattern is defined as the homogenous opacity of the bony canal, whereas the focal pattern was defined as the presence of one or more focal high-density areas; evaluating these peripheral medullary deposits on WBLD-CT is critical since they can be linked to high tumor burden, advanced disease stage, and poor prognosis in patients with symptomatic myeloma [18].

**Figure 11:**
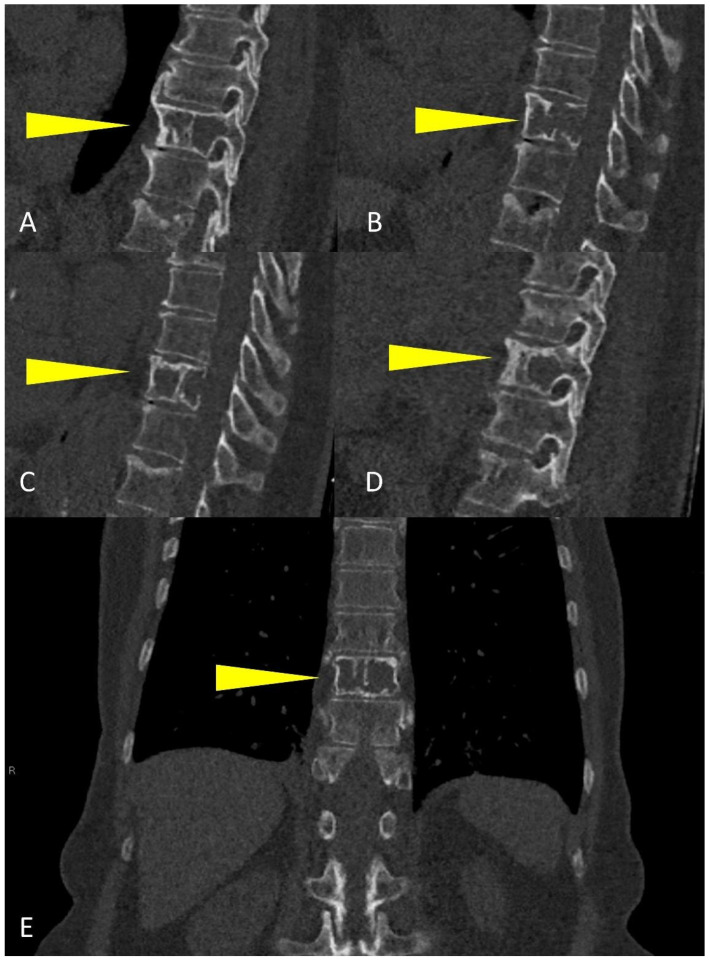
Sagittal CT reconstruction from right to left (**A**–**D**), and (**E**) coronal multiplanar reconstruction of a dorsal vertebra showing a large osteolytic lesion involving more than 50% of the vertebral body (yellow arrowheads), with high risk of fracture.

WBLD-CT is excellent in detecting fractures and their complications, such as vertebral compression, and in estimating fracture risk; the best plane for detecting vertebral fracture is the sagittal MPR view [15]. Vertebral compression fractures represent the most common type of fracture in patients with MM: common CT features in vertebral compression fractures are antero-lateral or posterior cortical fractures of the vertebral body, bone retropulsion, fracture lines in cancellous bone, and diffuse paravertebral soft tissue thickening; any form of destruction of cortical bone, cancellous bone or pedicle, focal paravertebral or epidural soft tissue mass indicates malignant vertebral compression fracture [33,34]. Vertebral fracture risk is usually classified according to the volume of osteolysis and its location within the vertebra: if more than 50% of the vertebral body is destroyed, involving critical parts (such as the costovertebral junction or the pedicle) the fracture is considered as high-risk and should be referred for prompt local treatment as stabilization; in case of spinal cord compression and/or foraminal compression, a neurological examination and a MRI should always be performed (Figure 11) [15].

## 4. Differentiation of Multiple Myeloma and Metastasis

Multiple myeloma and bone metastasis from other tumors can present with similar signs and symptoms (i.e., bone pain or pathological fractures) even though they are two largely different diseases. Lytic bone lesions are typical of both multiple myeloma and metastatic disease, whereas sclerotic bone lesions are most commonly seen in metastatic disease when compared to MM. These similarities in both symptoms and imaging presentation make these two pathological entities difficult to differentiate, even for very experienced radiologists. Dynamic contrast-enhanced MRI (DCE-MRI), diffusion-weighted imaging (DWI), and latest machine-learning techniques can be added to standard MRI examinations to help differentiate multiple myeloma from metastasis [35,36,37]. However, a robust contribution of clinical and laboratory information is still required nowadays to make a reliable differential diagnosis due to the massive overlap of imaging characteristics.

## 5. Report of Imaging Findings

A good and clear communication of imaging findings from the radiologist to the clinician is mandatory to ensure prompt management and treatment decisions: therefore, a structured report can be of great aid [15].

Every WBLD-CT report performed for MM should contain information on the used imaging technique (number of slices, slice thickness), on the presence of beam hardening or other artifacts. If any, confrontation with previous studies should always be performed.

MM-related findings should always be reported first, particularly bone findings (osteolytic lesions). Lesions report should follow an orderly fashion, the same as that used when evaluating CT axial images: starting from the skull to the spine, to the pelvic bones and the lower limbs; then upper limbs and ribcage bones are evaluated and, lastly, soft-tissue findings. Another reporting pattern could be based on lesion size, reporting firstly the biggest lesions, even though this approach may generate confusion in the reader. Size of the largest lesions should always be given. In case of focal or diffuse intra-medullary lesions, location, density and presence of endosteal scalloping must always be mentioned. Anyway, the presence of extensive osteolytic lesions or endosteal scalloping determines an increased risk of pathologic fracture (particularly on weight-bearing bones) and therefore should be stressed in the radiological report. In the case of pathological fractures involving the spine, with compression of spinal cord or other neural elements, the radiologist could suggest further MRI work-up, and should state whether the vertebral fracture is more likely to be malignant or not. Bone mineral density evaluation could be added to the report in order to give the clinician more information on the patient’s bones condition.

Incidental findings, as well, particularly the ones which could need further attention and evaluation, must find a place in every report [38].

A good report should contain a summary statement which highlights the main findings of the disease (number, distribution, size of the largest lesions) and every main or incidental urgent findings. Recommendation on follow-up examinations should be made.

## 6. Conclusions

Radiological whole-body techniques (whole-body low-dose CT, whole-body MRI, PET-CT) are nowadays deemed as mandatory and necessary for accurate diagnosis of multiple myeloma and for the evaluation of overall disease status. Thus, it is important for clinicians to define patient’s prognosis and to choose, therefore, the most appropriate treatment. In this context, whole-body low-dose CT has the advantage to better assess bone disease in patients suffering from monoclonal plasma cell disease, identifying osteolytic lesions that justify treatment in otherwise asymptomatic patients. This imaging modality is also useful to evaluate disease complications, such as pathological fractures, and to assess treatment response. For this purpose, the technical parameters of imaging acquisition and the patient’s position are essential to obtain good quality diagnostic images while maintaining a low radiation exposure. Nevertheless, it is important to adopt a systematic approach in image evaluation, which allows not to leave out any important feature and to avoid potential diagnostic pitfalls. Lastly, structured CT reporting should be used, as it favors reading of findings in order to better manage the patients and their pathology.
